# Assessing Demographic Characteristics, Microbiology, and Mortality Outcomes Associated With Vertebral Osteomyelitis: A National Single-Center Cohort Study in Malta

**DOI:** 10.7759/cureus.114615

**Published:** 2026-08-16

**Authors:** Matthew Spiteri, Simon Attard, Glenn Briffa, Neill Zammit, James Farrugia

**Affiliations:** 1 Internal Medicine, Mater Dei Hospital, Msida, MLT; 2 Medicine, Mater Dei Hospital, Msida, MLT; 3 Surgery, Mater Dei Hospital, Msida, MLT; 4 Neurosurgery, Mater Dei Hospital, Msida, MLT; 5 Infectious Diseases, Mater Dei Hospital, Msida, MLT

**Keywords:** cohort study, demographics, malta, microbiology, outcomes, spondylodiscitis, vertebral osteomyelitis

## Abstract

Background

Vertebral osteomyelitis (VO) is an uncommon but potentially debilitating spinal infection associated with delayed diagnosis, prolonged hospitalization, and significant morbidity. General international consensus indicates that the incidence of VO is increasing, which likely reflects an aging population and an increase in use of advanced imaging. This paper aims to describe the demographic characteristics, risk factors, microbiology, and short-term outcomes associated with VO in Malta.

Methods

We performed a retrospective single-centre cohort study of adult patients (>18 years old) diagnosed with VO at Mater Dei Hospital during the 2024 calendar year. Cases were identified through an internal VO registry. Since Mater Dei Hospital is the only national tertiary referral centre in Malta, the cohort is likely to be representative of the country's population. Medical records, electronic notes, imaging reports, and microbiology results were reviewed to extract the relevant clinical data.

Results

Twenty-three patients who were diagnosed with VO in 2024 were identified. The mean age was 63.1 years (range 27-87), and the majority of patients were male (69.6%). The mean length of admission was 39.3 days (range 3-153), with a median of 20 days, representing a significant hospitalization burden. The majority of cases involved the lumbar spine, with back pain being the most common presenting complaint. Associated risk factors and comorbidities included diabetes mellitus, chronic kidney disease, intravenous drug use, and preceding urinary tract infections. None of the patients had undergone prior spinal surgery or spinal procedures, consistent with a predominantly haematogenous pattern. A causative organism was identified in 19 cases (82.6%), with the most commonly isolated organism being *Staphylococcus aureus *in nine cases (39.1%). Three cases of streptococcal and two cases of enterococcalVOand*Mycobacterium* t*uberculosis *VO were identified. One case was due to a Gram-negative organism, and two cases with polymicrobial infection were identified. Complications included epidural, psoas, and paravertebral abscesses, disseminated infection, septic arthritis, and brain abscesses. The mortality rate was 17.4%, with two patients passing away within 30 days and two patients within one year.

Conclusion

In the Maltese cohort, VO predominantly affected older men with significant comorbidities, presenting most commonly as unexplained back pain with raised inflammatory markers. Other rarer presenting features included fever, weight loss, and neurological symptoms. The condition was associated with prolonged hospital stay and substantial infectious complications. Early recognition of high-risk patients may reduce diagnostic delay, morbidity, and healthcare burden.

## Introduction

Vertebral osteomyelitis (VO) is an infection of the vertebral body and the adjacent intervertebral disc. Although relatively rare, it is associated with considerable morbidity, typically due to non-specific symptoms, delayed treatment, and prolonged hospital stays [1-3]. Complications include sepsis, permanent neurological damage, and death. The condition typically affects the older population, and men are more likely to be affected [1-4]. Presentation is typically insidious and non-specific, characterized by back pain, fever, and elevated inflammatory markers (C-reactive protein (CRP) and erythrocyte sedimentation rate (ESR)) [2]. A range of comorbidities have been recognized as risk factors, including diabetes mellitus, intravenous (IV) drug use, malignancy, immunocompromised states, and chronic kidney disease [4]. Increased detection of these cases has likely been driven by improved access to advanced imaging techniques, such as magnetic resonance imaging (MRI) [5,6].

The majority of the currently available information regarding the presentation, microbiology, and etiology of VO that is currently available comes from large healthcare systems in Europe and North America, while smaller island states with a centralized referral pathway are underrepresented. To our knowledge, no previous epidemiological reports have been made regarding VO in the Maltese population; therefore, we sought to determine whether epidemiology in Malta reflects the well-established trends found in the broader literature. Our study aimed to describe the demographic profile, risk factors, microbiology, and short-term outcomes of VO in Malta over a single calendar year. Because Mater Dei Hospital is the sole tertiary referral centre on the island, it allows for the prompt involvement of infectious disease specialists, facilitating early input regarding relevant investigations, management, and comprehensive data collection.

## Materials and methods

Study design and case identification

A retrospective observational cohort study was conducted, including all patients diagnosed with VO in Malta between 1 January 2024 and 31 December 2024. Cases were reported by caring infectious disease teams in a prospective internal registry throughout the year. The diagnosis of VO was based on three factors: suggestive clinical features, supportive imaging, and microbiological or histological evidence. Following the 2015 Infectious Diseases Society of America (IDSA) guidelines, a diagnosis was made if an overall compatible clinical picture was present, combining these three factors [7]. An attempt was made to include all definite and probable cases of VO in individuals older than 18 years in the registry. It was assumed that all clinically relevant cases of VO were discussed with members of the infectious diseases team as per hospital protocol. However, as different specialists were reporting these cases throughout the year, information bias and selection bias could have occurred.

Data collection

Relevant data were extracted from online case notes, radiology reports, and microbiology laboratory results. Data were extracted consecutively by the authors into a common database and were periodically assessed by a data supervisor to prevent duplicate data and minimize inter-rater variability. Patient demographics were recorded, and the following categories were assessed: presenting complaints, relevant comorbidities and risk factors, any prior history of spinal surgery or lumbar puncture, imaging modalities used, spinal levels involved, isolated organisms and their culture source, duration of admission, and mortality within one year. Classical risk factors assessed included diabetes mellitus, IV drug use, immunosuppression or malignancy, chronic kidney disease, and preceding urinary tract infection. Throughout data collection and analysis, data governance and ethical considerations were observed. The use of data was approved by the hospital's Data Protection Office (DPO), and a specific ethics committee was not required. Patients were recruited in a consecutive manner to reduce selection bias. All data collected were anonymized and securely stored according to the hospital data protection policies and the General Data Protection Regulation (GDPR).

Data analysis

In view of the small sample size, descriptive analysis was used instead of inferential statistical testing. Continuous variables were reported as means, as well as interquartile ranges (IQRs), while categorical variables were expressed as frequencies and percentages.

## Results

Cohort characteristics

A total of 23 adult patients were identified and included in the study. The mean age was 63.1 years (range 27-87). The cohort was predominantly male, with 16 male patients (16/23; 69.6%) and seven female patients (7/23; 30.4%), giving a male-to-female ratio of approximately 2.3:1.

Clinical presentation

**Figure 1:**
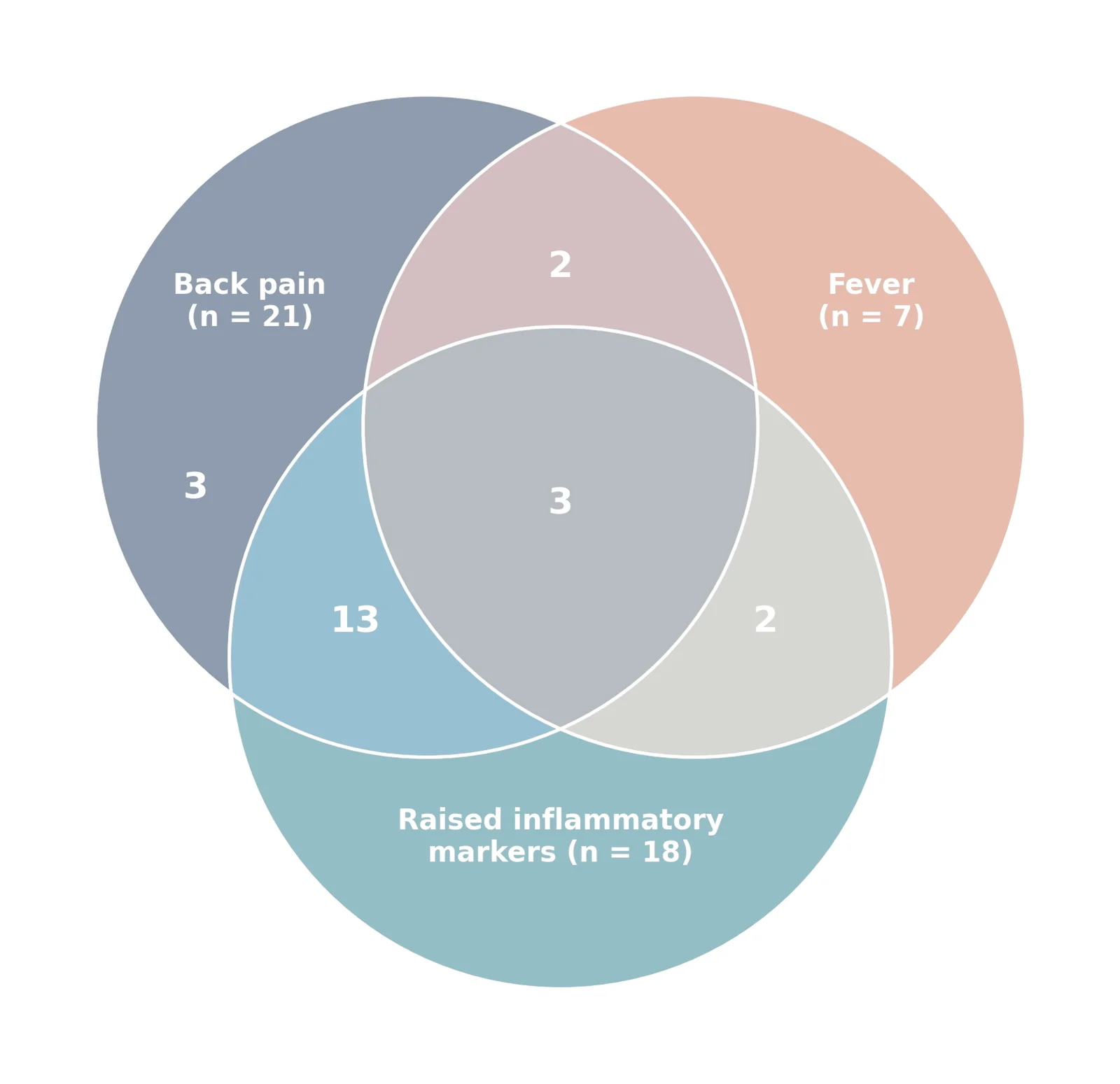
Key clinical phenotypes demonstrating the overlap of presenting features, including back pain (n = 21), fever (n = 7), and elevated inflammatory markers (n = 18). Numbers within overlapping regions indicate the number of patients exhibiting combinations of these presenting features.

The classical clinical presentation of back pain associated with raised inflammatory markers with or without fever was observed in 16 out of 23 cases (16/23; 69.6%). Only three patients presented with the full triad of back pain, fever, and raised inflammatory markers. The remaining patients presented with either one or two of these factors, as well as rarer presentations of neurological compromise or weight loss. The most common individual presenting feature was back pain, reported in 21 out of 23 patients (21/23; 91.3%). Fever was a presenting feature in seven cases (7/23; 30.4%). A neurological deficit as the presenting complaint was uncommon and seen in only one patient who presented with urinary retention and decreased lower limb reflexes. Weight loss was also uncommon and recorded in a single case (Figure 1).

Raised inflammatory markers were present in 18 cases (18/23; 78%), with a CRP median of 132.1 mg/L (IQR: 172.5, boundaries: 74.4 to 246.9) and an ESR median of 81.0 mm/hr (IQR: 46.3, boundaries: 56.3 to 102.5).

Risk factors

**Figure 2:**
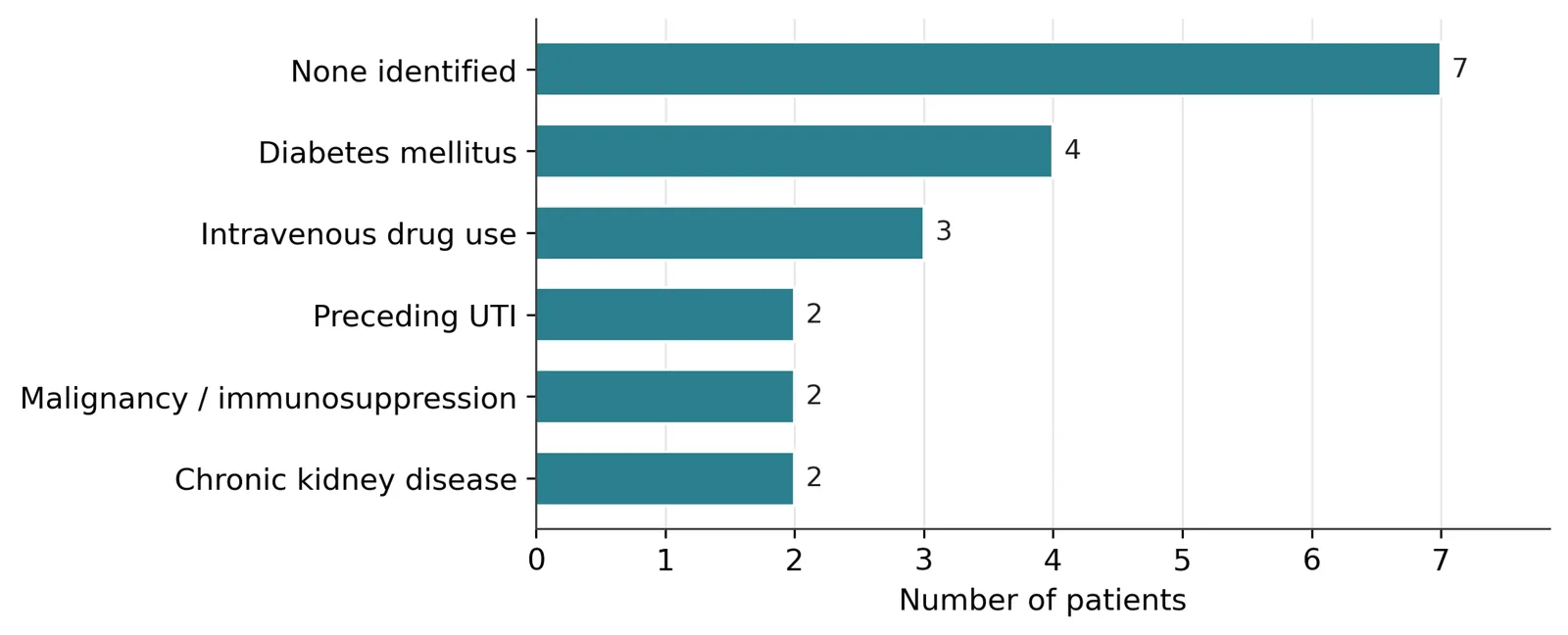
Comorbidities and predisposing risk factors. UTI: urinary tract infection

At least one classical risk factor was present in the majority of cases, occurring in 16 out of 23 patients (16/23; 69.6%), whereas seven patients had no identifiable risk factor (7/23; 30.4%). The most commonly associated risk factor was diabetes mellitus, observed in four patients, followed by IV drug use, noted in three patients. Preceding or ongoing urinary tract infections, concurrent malignancies or immunosuppressive states, and chronic kidney disease were each present in two patients. Notably, none of the patients had a history of prior spinal surgery or lumbar puncture (Figure 2).

Site of involvement

**Figure 3:**
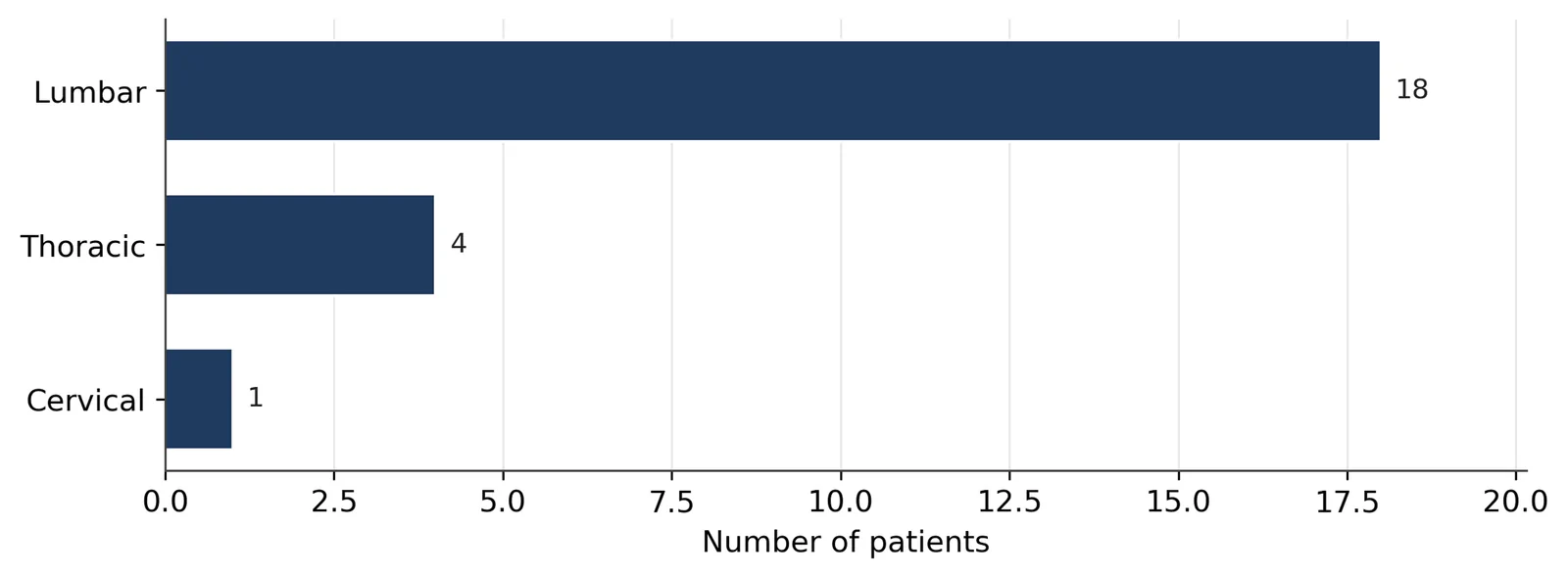
Anatomical distribution of spinal involvement.

The most common site involved was the lumbar spine, with 18 patients (18/23; 78.3%) affected, followed by thoracic spine disease in four patients (4/23; 17.4%) and cervical spine involvement seen in one patient (1/23; 4.3%) (Figure 3).

Microbiology and investigations

A causative organism was identified in 19 out of 23 cases (19/23; 82.6%). Regarding the diagnostic pathway, blood cultures were utilized as the first-line investigation. In cases where blood samples isolated an organism clinically consistent with VO, this isolate was designated as the causative pathogen. This occurred in 15 out of the 19 culture-positive cases.

The following organisms were identified by blood cultures as causative pathogens: *Staphylococcus aureus* (eight cases), *Enterococcus faecalis* (two cases), *Citrobacter koseri* (one case), *Streptococcus pneumoniae* (one case), *Streptococcus sanguinis* (one case), co-infection with *S. aureus* and *Streptococcus agalactiae* (one case), co-infection with *Streptococcus salivarius* and *Bacteroides fragilis* (one case).

In five of these 15 cases, additional diagnostic certainty was provided by positive cultures obtained from secondary sample sources: pus aspirate from localized collections (two cases), bone biopsy (one case), a wound swab from a contiguous sacral ulcer (one case), and vitreous humor in a patient with concurrent endophthalmitis (one case). These secondary investigations were carried out concurrently with or prior to the finalization of blood culture results.

In the four cases where blood cultures were negative (4/23; 17.4%), secondary investigations successfully identified a pathogen. A bone biopsy revealed *S. aureus* in one case, while pus aspirate from associated collections identified two cases of *Mycobacterium tuberculosis *and one case of *Streptococcus dysgalactiae*.

**Figure 4:**
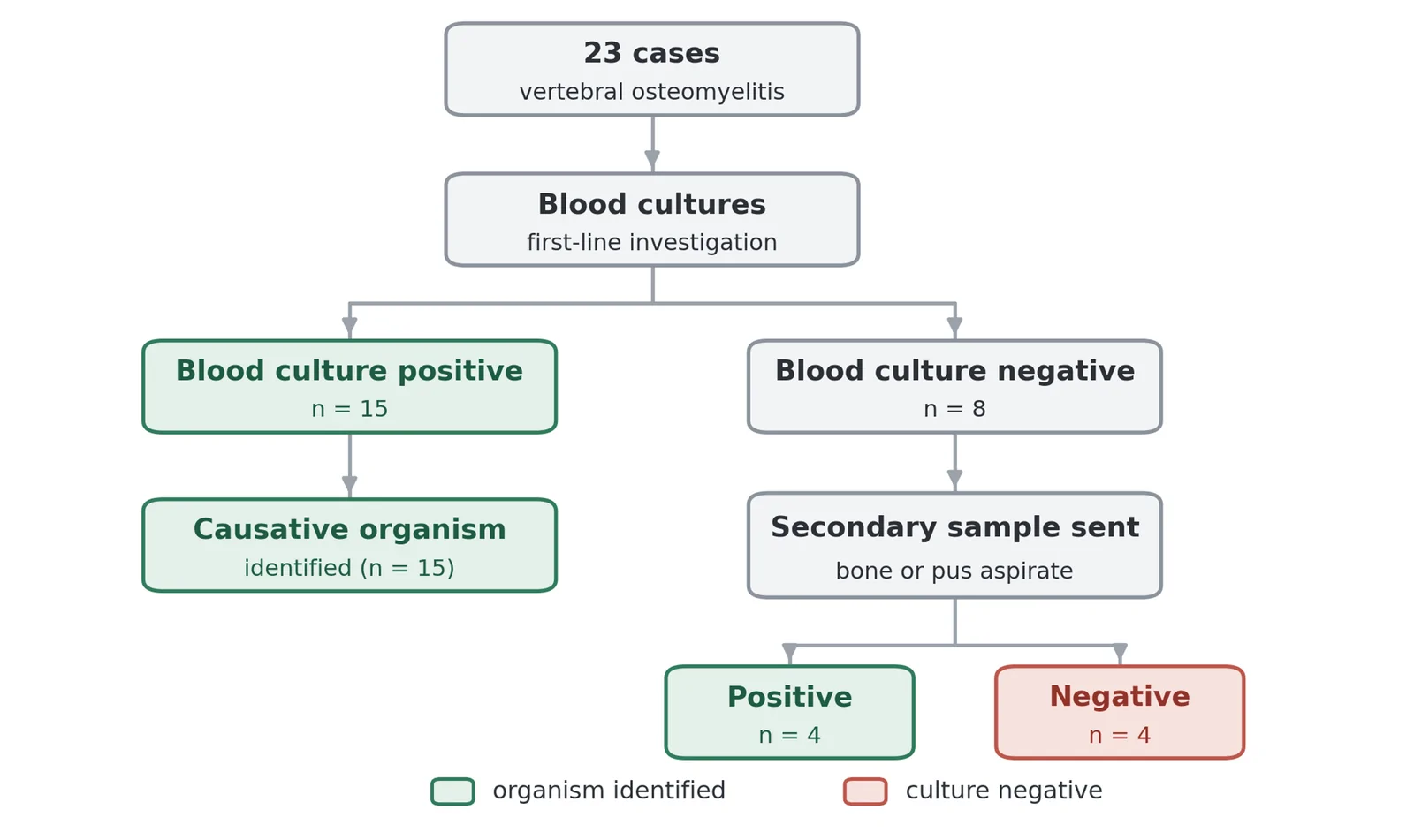
Microbiological investigation pathway. Blood cultures were the first-line investigation; blood-negative cases proceeded to secondary bone or pus sampling.

The remaining four cases were deemed culture-negative. Among these, one case demonstrated no growth on both blood and bone samples. In two cases, no organism was cultured from blood samples, and a bone biopsy was not performed for clinical reasons. In one case, a single blood sample was positive but was interpreted as a contaminated skin commensal (*Staphylococcus epidermidis*), and subsequent bone cultures were negative (Figure 4). Of the four culture-negative cases, two patients were receiving broad-spectrum antibiotics prior to initial investigations.

**Figure 5:**
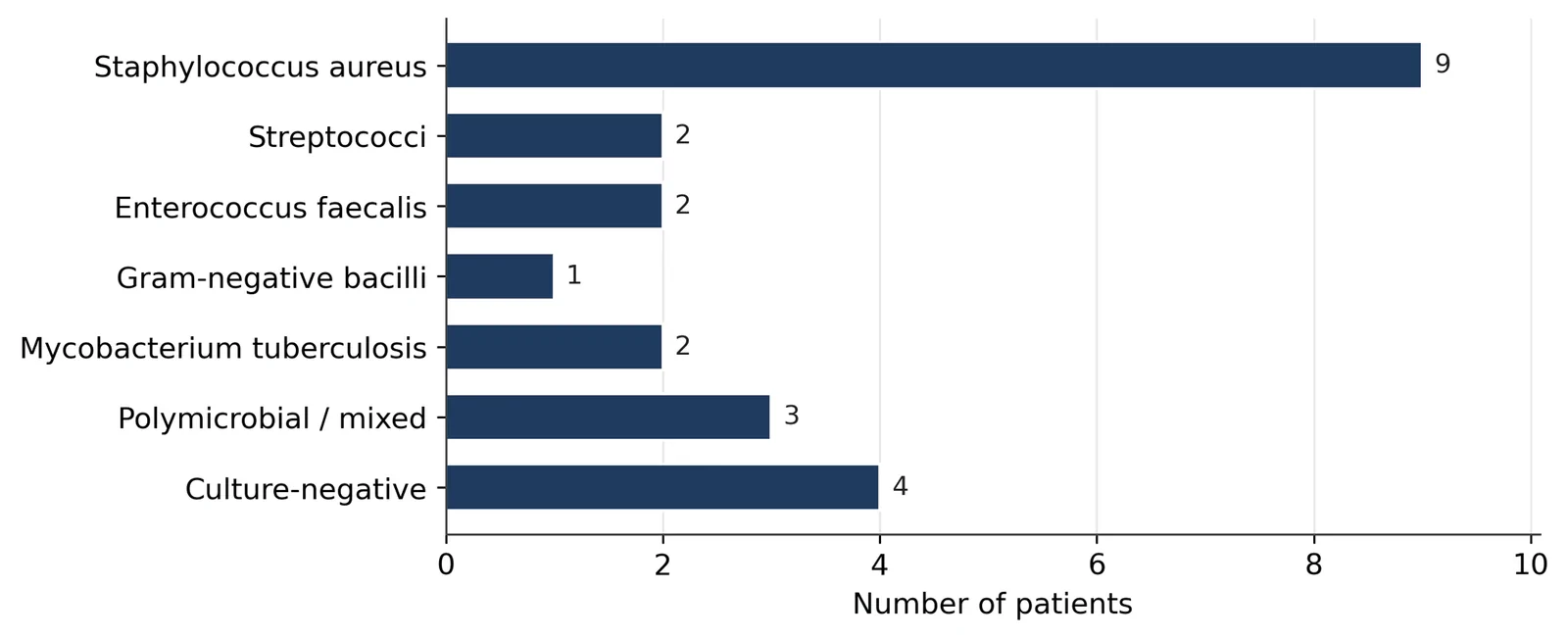
Isolated organisms grouped by category. A causative organism was identified in 19/23 cases; four were culture-negative.

Regarding pathogen distribution, *S. aureus* was the most frequently isolated organism, identified as the sole pathogen in nine cases (9/23; 39.1%); eight isolates were methicillin-sensitive *S. aureus* (MSSA), and one was methicillin-resistant *S. aureus* (MRSA). A further case yielded MSSA co-isolated with *S. agalactiae* and was classified among the polymicrobial infections. Monomicrobial infections with *E. faecalis* and *M. tuberculosis* were each identified in two patients (2/23; 8.7%). Streptococcal monomicrobial infections were identified in three cases, with isolates of *S. pneumoniae, S. dysgalactiae,* and *S. sanguinis* identified. One polymicrobial facet-joint infection yielded *S. salivarius* and *B. fragilis*. The Gram-negative pathogen *C. koseri* was identified in a single case (Figure 5).

Imaging modalities used included MRI, computed tomography (CT), and positron emission tomography (PET). In several cases, more than one modality was used to confirm the diagnosis and assess for complications.

Outcomes

The mean duration of admission was 39.3 days (range 3-153), with a median of 20 days and an IQR of 26 days. This represents a substantial inpatient healthcare burden. Several cases presented with relatively advanced disease complicated by spinal fractures or abscesses. Twelve out of 23 cases (12/23; 52.2%) demonstrated some form of paraspinal or extradural pus collection, often in multiple locations. One case presented with disseminated MSSA involving cerebral, epidural, and psoas collections. Another case was complicated by a wedge fracture and destructive osseous changes. This high proportion of cases with extensive disease exerted a significant impact on hospital resources and length of stay.

The one-year all-cause mortality rate was 17.4% (4/23), with two patients passing away within 30 days and two within one year. A summary of the key findings can be found in Table 1.

**Table 1:** Summary of demographic, microbiological, and outcome data for the cohort (n = 23). IQR: interquartile range; CRP: C-reactive protein; ESR: erythrocyte sedimentation rate

Variable	Result
Number of cases	23
Mean age, years (range)	63.1 (27-87)
Male sex, n (%)	16/23 (69.6)
Female sex, n (%)	7/23 (30.4)
Mean length of admission, days (range)	39 (3-153)
Median length of admission, days (IQR)	20 (IQR 26 17-43)
Most common spinal region, n (%)	Lumbar (18/23, 78.3)
Most common organism, n (%)	*Staphylococcus aureus* (9/23, 39.1)
Most common phenotype	Back pain + raised inflammatory markers
Pathogen identified, n (%)	19/23 (82.6)
IQR for CRP (all 23 cases)	172.5, boundaries: 74.4 to 246.9
IQR for ESR (checked in 16 cases)	46.3, boundaries: 56.3 to 102.5
Prior spinal surgery/procedure	None reported
One-year all-cause mortality, n (%)	4/23 (17.4)
Maltese nationality, n (%)	19/23 (82.6)
Non-Maltese nationality, n (%)	4/23 (17.4)

## Discussion

This single-center case series provides the first description of VO in the Maltese population. The demographic profile presented here aligns closely with broader patterns reported in international literature regarding larger healthcare systems. The condition predominantly affected older adults with a mean age of 63.1 years and was more common in men, with a male-to-female ratio of 2.3:1. This reflects the age and sex distributions described consistently in other European, North American, and Asian studies [1,4,6,8], confirming that despite Malta's small population, the epidemiology of the disease remains consistent.

The predominant presenting phenotype of our cohort was consistent with international findings, with back pain and raised inflammatory markers, with or without fever, being the most common presentation [8,9]. Specifically, 21 out of 23 cases reported back pain, 16 of whom were noted to have raised inflammatory markers, and only seven of whom had reported febrile episodes. This highlights the fact that fever is not a highly specific marker for VO, and its absence should not mislead the clinician away from the diagnosis [10]. Neurological compromise was only found to be a presenting feature in a single case.

The comorbidity profile was also in keeping with other published sources, with diabetes mellitus being the most common association [2,4]. This was followed by IV drug use and ongoing urinary tract infections. It is important to note that in one-third of cases, no recognized risk factor was identified. This emphasizes the importance of maintaining VO as a differential diagnosis even in the absence of associated comorbidities or obvious vulnerability, especially in patients with unexplained back pain and raised inflammatory markers. In one case, a contiguous sacral sore was identified; however, there were no cases with prior surgical procedures or instrumentation indicating that hematogenous spread was the predominant route of infection in our cohort [11].

The microbiological picture reflected established international trends, with *S. aureus* being the leading causative organism [8,9]. This provides valuable therapeutic insight when the need arises to prescribe empiric treatment. The 2015 IDSA guidelines and international expertise advise withholding empiric treatment until diagnostic samples are obtained, except in cases of sepsis or neurological compromise [7], where priority is given to immediate broad-spectrum antimicrobial therapy. An important consideration is the possibility of spinal tuberculosis (TB), particularly in cases of foreign origin. Latent TB infection in the Maltese population is thought to be very low, but is notably higher in the immigrant population. Both TB cases in our study occurred in non-Maltese individuals. In such cases, standard blood cultures were negative, with the microbiological diagnosis made from mycobacterial cultures of pus samples.

Our cohort demonstrated a high percentage of organism identification, with a microbiological diagnosis made in 19 out of 23 cases. When compared to other series, this percentage lies towards the higher end of the reported spectrum [8,10]. Several factors may explain this finding based on the local healthcare structure. With Mater Dei Hospital being the sole tertiary healthcare center in Malta, infectious disease specialists were involved early and consistently, allowing for prompt advice regarding appropriate investigation pathways and limiting use of unwarranted empirical antimicrobial therapy. Fifteen diagnoses were made via blood cultures, with secondary sampling recovering an organism in a further four cases. These four cases reflect the ease of access to a range of resources and multidisciplinary input available at Mater Dei Hospital. Time-sensitive, invasive procedures such as bone biopsies and image-guided aspirations could be organized rapidly within the same center [7]. Out of the four culture-negative cases, two were receiving broad-spectrum antibiotics prior to samples being taken.

The burden on the healthcare system caused by this condition is undeniable, with a mean inpatient stay of almost 40 days. However, looking at the median of 20 days with an IQR of 26 days (17 to 43) provides a deeper insight into the factors affecting hospital duration. Out of 23 cases, half were discharged in less than 20 days, with the average duration being heavily swayed by two particularly complicated cases that required more than 100 days of inpatient care. This relatively short duration of stay for the remaining patients could reflect that within this time period, the relevant investigations and sensitivities were obtained, a formal diagnosis was made, and where possible, antimicrobials were rationalized to oral therapy [12]. In cases that were culture-negative, or had resistant organisms requiring prolonged IV empiric therapy, patients could still be safely discharged home via the Home Antibiotics Therapy (HAT) service provided by Mater Dei Hospital.

There were several limitations that temper these observations. This was a retrospective study focusing on a single calendar year. The cohort size of 23 is adequate to provide descriptive analysis but too small to support inferential analysis or identify predictors of poor outcome. Long-term clinical outcomes, beyond all-cause mortality at one year, were not assessed in this study. Data were drawn retrospectively from self-reported cases registered by a number of different medical professionals throughout the year, which could have led to selection and information bias. Prospective, multi-year follow-up studies through the existing registry would allow incidence trends and outcome determinants to be examined more robustly, and would make it possible to quantify the impact of the diagnostic and community-treatment pathways described here.

## Conclusions

This first national cohort study confirms that VO in Malta mirrors the international picture in terms of epidemiology, clinical presentation, and microbiology. It predominantly affects older comorbid men, commonly involves the lumbar region, and typically spreads via the hematogenous route. Regarding the presenting phenotype, unexplained back pain in the presence of raised inflammatory markers should prompt spinal imaging even in the absence of fever or associated risk factors. Empiric antimicrobials should be withheld until adequate diagnostic samples are obtained, provided the patient's hemodynamic stability and clinical status safely allow. Moving forward, larger prospective studies would be beneficial to assess long-term functional outcomes.
